# Understanding the factors that influence matriculation and persistence in Black medical students

**DOI:** 10.3389/fmed.2023.1189666

**Published:** 2023-05-18

**Authors:** Krista D. Mincey, Briana S. L. Richardson, Raymond O. Johnson, Mary L. Heraut

**Affiliations:** ^1^Department of Community Medicine, Mercer University School of Medicine, Macon, GA, United States; ^2^First-Year Medical Student, Mercer University School of Medicine, Columbus, GA, United States; ^3^First-Year Medical Student, Mercer University School of Medicine, Savannah, GA, United States; ^4^Department of Public Health, College of Health Professions, Mercer University, Atlanta, GA, United States

**Keywords:** qualitative, medical students, matriculation, persistence, Black/African American

## Abstract

**Purpose:**

The Association of American Medical Colleges’ (AAMC) Strategic Plan lists 10 action plans one of which is focused on understanding how systemic barriers, such as racism and access to quality education, may negatively impact diversity in academic medicine. Thus, the purpose of this study was to understand the factors that impact the matriculation and persistence of Black medical students.

**Method:**

A qualitative phenomenological study using Tinto’s Model of Institutional Departure as an organizing framework was used for this study. Participants were asked a series of questions covering topics related to their goals, their medical school experience, their preparation for medical school, what could improve their medical school experience, and advice for future Black medical students.

**Results:**

Forty in-depth semi-structured interviews were conducted during the fall 2022 term from October to December with Black medical students enrolled in over 16 US or Caribbean medical schools. Findings reported that two factors impacted matriculation for Black medical students (exposure to the medical field and resources, particularly financial resources). Findings also reported that three factors impacted the persistence of Black students once in medical school (diversity, support, and emotional resources).

**Conclusion:**

The five factors identified by participants that impact matriculation and persistence for Black medical students can be used by medical schools to increase their enrollment and graduation of Black students.

## Introduction

Although America’s population has trended toward diversity, statistics show that the physician workforce is a poor reflection of the diverse population it strives to serve. Specifically, Black Americans account for over 13% of America’s population yet only 5% of physicians are Black (1). These statistics become even more concerning considering that the population of Black male physicians has trended downwards since 1978 and now Black men only account for 3% of all physicians (2, 3). Research has asserted the importance of racial/ethnic concordance in addressing health disparities and patient satisfaction (4, 5). And the COVID pandemic further highlighted how the physician workforce statistics correlate to greater health disparities and worsened outcomes (6, 7). With a projected shortage of physicians by the year 2034, now is the time to research solutions that will prevent a greater contrast between America’s population and the physician workforce. Some medical experts believe that diversifying the physician workforce is an integral part of the solution to addressing the physician shortage altogether (1). However, increasing the population of Black physicians is preceded by increasing the matriculation and graduation rates of Black students through medical school.

Although 2021 AAMC data reported a 20% increase in first-year matriculants, data also indicate a variation between the matriculation of Black students and the persistence of Black students in medical school (8–10). Black student attrition rates more than double the attrition rates of white students, and studies show that even if Black matriculation rates were tripled, the time needed to correct the deficit of Black physicians would equate to more than 20 years (10, 11). Why are Black students less likely to graduate medical school than their peers? The answer to this question could help medical schools address both the physician shortage and the lack of Black physicians in the physician workforce by increasing Black student matriculation rates and decreasing the attrition rates of Black students. Therefore, it is important for research to address the barriers that Black students face while matriculating through medical school. Thus, the following research question guided this study: What factors impact matriculation and persistence in medical school for Black students?

## Materials and methods

### Design

This study employed a qualitative design with in-depth interviews using the approach of phenomenology. Phenomenology is a qualitative approach that describes “the common meaning for several individuals of their lived experiences of a concept or a phenomenon” (12, p. 75). Additionally, interview questions for this study were guided by Tinto’s Model of Institutional Departure. Before recruitment and data collection began, this study received IRB approval from Mercer University.

To describe matriculation and persistence among Black medical students, semi-structured in-depth interviews were used. Black medical students were recruited to participate in this study through email. The first author sent an email to Deans, Directors, and coordinators in the offices of Diversity & Inclusion, Admissions & Recruitment, and Multicultural Affairs at medical schools throughout the US explaining the study, providing a recruitment flyer, and asking them to send it out to students. Participants were also recruited by the first author sending the recruitment flyer to medical students they knew and asking them to send the information to medical students. Inclusion criteria for participation in the study were: (a) currently enrolled in a medical school in the United States or the Caribbean, and (b) self-identify as African American or Black. Recruitment and interviews took place during fall 2022.

Persons who were interested in participating in the study emailed the first author expressing interest in the study. At that time, they were sent a Google form with interview dates and times and asked to select one date and time from the list. After they signed up for an interview date, they were sent a calendar invite with Zoom information for the interview and the informed consent document. Participants were also sent a reminder email before their interview with the Zoom information.

### Procedure

The first author who is mainly a qualitative researcher conducted all interviews. Interviews lasted no more than 45 min. Interview questions were guided by Tinto’s Model of Institutional Departure (13). The model suggests that students have to be integrated into the social and academic system of their institution in order to maintain at that institution (13). The model suggests that students come into college with attributes (family background, skills abilities, prior schooling) that develop their goals and commitments (13). Once they engage in the social (activities and peers) and the academic (faculty/staff and their performance) environments at their college and become integrated, their goals and commitments may change which then determine if they stay at their college (13). The model further suggests that a student’s external commitments to things such as family and work impact their goals and commitments which can ultimately influence whether they stay in school (13). For the purposes of this research, interview questions were developed around what impacted their preparedness for medical school, their goals, their connection to their medical school, their suggested improvements for their medical school, and their advice for future Black medical students. Interview questions were developed by the first author and were not reviewed by other researchers or student researchers. A list of interview questions is in Table 1.

**Table 1:** Interview questions.

How did your family impact your decision to pursue medical school?
How academically prepared do you believe you were for medical school?
How do you believe your race influenced your preparedness for medical school?
How do you believe your gender influenced your preparedness for medical school?
What goals did you have coming into medical school? (personal, academic, professional)
What goals do you have now that you are in medical school?
What connection do you have with your medical school?
What connection do you have with the faculty at your institution?
What connection do you have with your classmates at your institution?
What could be done to improve these connections?
How do your non-school connections (family, friends, partner) impact your work in medical school?
Have you faced any difficulties in medical school?
Have you had positive experiences in medical school?
What would make your medical school experience better?
What advice would you give future Black medical students?

Before all interviews began, participants were asked if they consented to have the interview recorded. Once they consented to the interview being recorded, informed consent was explained and obtained via Zoom recording. After informed consent was obtained, participants were asked a series of demographic questions before the interview started. Zoom recordings of interviews were deleted after the analysis was conducted. Participants were provided a $50 gift card for their participation.

### Analysis

All interviews were recorded via Zoom and transcribed using a transcription service. Through the use of deductive codes, the transcripts were analyzed using Dedoose software. To develop the deductive codes, authors 2–4 were assigned a certain number of transcripts and asked to develop codes along with definitions for elements that appeared frequently in the transcripts they were assigned. Once codes and definitions were developed, they were reviewed by the first author for clarity before using the codes to analyze the transcripts using Dedoose software. A determination of overall themes was initially determined by looking at codes that appeared in at least 50% of all transcripts which came out to three general themes (support, resources, and emotional). Once this was completed, the co-occurrence among codes was assessed with support and resources having the most co-occurrences across all transcripts. From this analysis, findings were developed and sent to participants for member checking to see if participants agreed with the findings and had edits or additions to the findings.

## Results

There were 40 participants ranging from 22 to 40 years of age. Ninety percent of participants were enrolled in an MD program, while 10% were enrolled in a DO program. Almost half of the participants (48.8%) were 25 to 27 years of age (Table 2). More than half of the participants were female (65.9%). Among all participants, more than half were in their first year of medical school (73.2%). Half or more than half of the participant’s mothers (57.5%) and fathers (50%) were college graduates or had a professional degree. Sixty-one percent of all participants were single with most single participants being female (77.8%). Most participants were paying for medical school through loans or scholarships or a mix of both. All male participants (14) were attending medical school at a Predominately White Institution (PWI). Among female participants, 11.1% of participants were attending medical school at a Historically Black College and University (HBCU). Table 3 lists the specialties the participants are planning to go into. While four participants listed more than one specialty area, the top three specialties that participants are planning to go into are (a) primary care specialty (12), (b) surgery specialty (9), and Dermatology (5).

**Table 2:** Characteristics of interview participants.

Characteristics	Total (*N* = 41)	Male	Female
	*n* (%)	14 (34.2%)	27 (65.9%)
*Age*
22–24	11 (26.8%)	6 (42.9%)	5 (18.5%)
25–27	20 (48.8%)	6 (42.9%)	14 (51.9%)
28–30	6 (14.6%)	1 (7.1%)	5 (18.5%)
31–33	3 (7.3%)	1 (7.1%)	2 (7.4%)
34–36	–	–	–
37–40	1 (2.4%)	0	1 (3.7%)
*Year in medical school*
First Year	30 (73.2%)	12 (85.7%)	18 (66.7%)
Second Year	5 (12.2%)	1 (7.1%)	4 (14.8%)
Third Year	3 (7.3%)	1 (7.1%)	2 (7.4%)
Fourth Year	3 (7.3%)	–	3 (11.1%)
*Paying for school (could provide more than one option)*
Work part-time
Work full-time
On scholarship	21	10	11
Loans	34	10	24
Pell grant			
Work-study			
Other			
GI Bill	2	2	-
Savings	2	–	2
Parental support	1	–	1
*Mother’s education*
8th grade or less
Part high school
High school graduate	7 (17.1%)	1 (7.1%)	6 (22.2%)
GED	2 (4.9%)	1 (7.1%)	1 (3.7%)
High school graduate plus vocational training	2 (4.9%)	1 (7.1%)	1 (3.7%)
Part college	7 (17.1%)	1 (7.1%)	6 (22.2%)
College graduate	10 (24.4%)	3 (21.4%)	7 (25.9%)
Graduate or professional degree	13 (31.7%)	7 (50%)	6 (22.2%)
*Father’s education*
8th grade or less
Part high school	1 (2.4%)	–	1 (3.7%)
High school graduate	3 (7.3%)	–	3 (11.1%)
GED	2 (4.9%)	1 (7.1%)	1 (3.7%)
High school graduate plus vocational training	1 (2.4%)	1 (7.1%)	–
Part college	6 (14.6%)	2 (14.3%)	4 (14.8%)
College graduate	7 (17.1%)	3 (21.4%)	4 (14.8%)
Grad/Professional degree	13 (31.7%)	5 (35.7%)	8 (29.6%)
Unknown/not applicable	8 (19.5%)	2 (14.3%)	6 (22.2%)
*Relationship status*
Single	25 (61%)	4 (28.6%)	21 (77.8%)
In a relationship	13 (31.7%)	8 (57.1%)	5 (18.5%)
Married/Partnered	3 (7.3%)	2 (14.3%)	1 (3.7%)
Separated/Divorced	–	–	–
*Type of school*
PWI	37 (90.2%)	14 (100%)	23 (85.2%)
HBCU	3 (7.3%)	–	3 (11.1%)
Caribbean	1 (2.4%)	–	1 (3.7%)

**Table 3:** Specialty participants are planning to go Into.

Specialty	Total (*N* = 40)	Male	Female
Primary care (primary care, pediatrics, internal medicine, family medicine)	12^+^	4	8
Anesthesiology	3	2	1
Orthopedics	2	1	1
Surgery (general, neurosurgery, surgical specialty, trauma)	9	4	5
Dermatology	5^^^	–	5
Emergency medicine	2	1	1
Psychiatry	2	1	1
Obstetrics/Gynecology	4^+^	–	4
Unsure	1	–	1
Ophthalmology	1^^^	–	1
Otolaryngology	1	1	-
Pathology	1	–	1
Oncology	1	–	1

+ One participant selected ob/gyn and Pediatrics.

^ One participant selected Ophthalmology and Dermatology.

### Factors influencing matriculation and persistence

For this study, we are defining matriculation as the process a person goes through to get accepted into medical school. We are defining persistence as a person’s ability to maintain through difficult moments once in medical school in order to get to graduation. Participants’ responses to factors that impact their matriculation and persistence in medical school can be separated into five areas: exposure, resources, diversity, support, and emotional. Overall, participants’ responses appeared to reflect that exposure and resources are important factors for their matriculation into medical school; however, diversity, support, and emotional factors are influential to their persistence in medical school.

#### Pre-entry attributes

Pre-entry attributes are factors that participants have or have experienced before coming to school. The two factors associated with this are exposure and resources.

### Exposure

Exposure was related to not growing up being exposed to someone in medicine. Participants mentioned how not having this kind of access caused them to work harder to find out information that they needed to apply to medical school or to find other resources they needed to apply to medical school. As mentioned by two participants.

Most of my classmates are children of physicians and if they are not children of physicians they – you know, somebody in the family is a physician. Some of them are like sixth generation doctors, you know, so with that comes a level of being put on game that I do not think I had….So I think there’s a lot of insight that is reserved for people who have access to the inside of medicine early on and usually it’s not people who look like me, so I feel like when I found the classmates who are Black or of color who have parents in medicine they are few and far in between.

…literally every step of the way, like I do not have, you know, my – my dad, whose name is Tom, who has, you know, been a doctor for generations, I cannot just go like shadow at my mom’s office. I had to literally not only like follow a path, but literally create a path and hope that it was like going in the right direction.

### Resources

Resources were related to things participants needed to help progress toward their goals. Participants mentioned how a lack of resources hindered their preparation for medical school. As mentioned by one participant, “…MCAT was my biggest holdback, because I did not have thousands of dollars for a Kaplan course, so I did a lot of self-study. I took the MCAT three times. And that definitely held me back.”

Participants also mentioned how having access to resources was helpful for their medical school preparation. As mentioned by two participants.

I was thankful to have known a couple of Black physicians going in, and that was definitely helpful. Being able to talk to them, like know what it’s like, like see where like things I needed to work on, things I did not need to work, where like economically I realized that I would need more help, I got most of that advice from Black physicians.

…I think that the reason why I was able to even prepare for med school was because of my race. Like I qualify for the pay assistance program through the AAMC, so I did not have to pay for any of my primaries that I sent, and that was like a really big help, and I know like, you know, not even just my race, but also my like socioeconomic status helped with that.

#### Institutional experience

Institutional experience deals with the factors related to the experience participants have with their institution, their classmates, and the faculty and staff. The three factors associated with this are diversity, support, and resources.

### Diversity

Diversity was related to the importance of having diversity reflected at their medical school. Participants understood the importance of diversity and wanted there to be more strategic efforts by their schools to have diversity. As mentioned by two participants.

…just more diversity. I feel like I really bonded with the previous class because they have more Black folks. They had like 15 Black men, 20 Black women to our like eight Black men, five Black women, but I think just more diversity specifically, yeah, that would make me more happier hanging out with more people like me.

…what would make my experience better…I would say more critical mass, and what I mean by that is it does not necessarily have to be more African-American students, but it’s just more people that I have things in common with. I think that always makes the experience great, because you do not feel so isolated, you know, feel so bogged down with school.

While participants wanted more diversity, some also mentioned that their schools have made some changes toward diversity, but that more efforts were needed. As mentioned by one participant.

…they have been trying to put an emphasis on, you know, the diversity and equity and inclusion and like making sure our curriculum is supporting those efforts…I definitely know that it has improved since, you know, X number of years when they started doing these things, but there is a lot of room for growth…I would love for treating patients of color to be better than it is…I think the avenues to get those changes made are very, very frustrating…that it has not happened yet because it’s so difficult to make those changes…

### Support

Support was related to having assistance or someone to lean on when facing difficult times. Participants mentioned how having support from all leadership levels at their school was important to their success along with having support from classmates. One participant mentioned how having support pushes them to succeed.

…I definitely know that when you have that like support system from like, you know, people with higher powers or higher positions than you that want you to succeed, like – and even though they say it, whenever they show it, you know, through things like meetings, one on one meetings, yeah, stuff like that definitely helps you to feel like, you know, it’s more – there’s more kind of pressure, but like motivating pressure on you to succeed, because now it’s not only about you.

Another participant mentioned how having support would have helped them academically.

…they have dropped the ball with me and everybody else who has been in my same position because there are a lot of us. And they – most of us look like me, who have been through this of like failing classes and like struggling through, you know, board exams. I just – if you saw that there was a problem I feel like you should have intervened sooner and offered me something else from what everybody else is doing, because obviously what everybody else is doing is not working, so that would have helped a lot.

Another participant spoke about the importance of having support from other Black students for their success.

They have provided opportunities for first-year medical students to kind of engage and interact with upperclassmen who are underrepresented in medicine, which has been great because we have been able to establish relationships with some M2s, which is very, very helpful in navigating.

Another participant spoke to why having support from classmates is critical for Black medical students, “It’s already hard being the minority, (laughs) but yeah, having people that, you know, relate to on a deeper level, you know, culturally definitely helps out a lot. It’s – Yeah, much easier to talk to them.”

### Resources

Resources were related to things participants needed to help progress toward their goals. Participants mentioned how having support from their school along with tangible resources would help them while in medical school. As mentioned by one participant.

…I think a lot of schools do this where they recruit students of color just like you know, recruit them, and then once they get here it’s kind of just like they just drop you off and like there’s no support once those students get here.

Other participants mentioned how having access to tangible resources is important to their success in medical school.

Office of multicultural affairs is so supportive. Like they have like books…like everybody has access to an electronic copy, but if you want the book version, you can just go to the office and see if they have it, and then you can borrow it, and your only situation is like when you are done you can give it back so another student can use it next year, which I think is very lovely. Just like that, kind of like community has been really nice.

…wellness has been one thing that us as a class have really wanted to be addressed better is feeling like the workload is high, but like we are at a point where like mental health resources and just the state of mental health for a lot of medical students, especially those who are entering during the pandemic is at like – it’s really suffering, and so we need the resources, the time, the space to unwind.

…it would be nice to actually have a real school building…I’ve been to like other med schools…and they have an actual building and the students are there. You see them all the time. They have different floors, and it’s just for them. We have like a floor on one big building and then like a floor on another big building. It’s –…it’s just not enough.

#### External community

External community deals with factors related to the participants’ family, work, and other commitments. The two factors associated with this are support and emotional.

### Support

Support was related to having assistance or someone to lean on when facing difficult times. Participants mentioned how having tangible support from their family and friends was critical to them being able to be in medical school. As mentioned by one participant.

…my family is my – They’re a village. Like I really do have a village. I have my family – when I went through some financial troubles…all of my refund was gone, so…my parents took over trying to pay wherever they could. I’ve had my friends send me gas money…

Another participant mentioned the importance of tangible support from their family.

My parents, they help cover the living expenses, mainly rent, so that’s not a financial hurdle that I have to worry about for right now. And they helped me move in. They helped get a lot of things for me when I moved in here, like bed, dresser, night stand, another monitor so I do not have to stare at everything on my laptop. So they really helped me there.

Even if a participant’s family could not provide tangible support, they mentioned they were still able to provide emotional support which was just as important.

### Emotional

Emotional was related to support from family, friends, or partner that you can lean on who keep you level-headed.

…just being able to like have someone to talk to I think is really important, who is not in medicine – or not in med school with you rather. Yeah, I think that’s really important just to pull you back from all the studying your doing and just remind you that you are an actual person, and that you can enjoy some personal time, so yeah.

…So…family-wise…they are very supportive. Usually call them a couple times a week or once a week, know they are in my corner, so that’s just a very good thing to have, just – yeah, a level of support that helps me do well in school…

## Discussion

Although diversity within medical schools has increased over the years, there has been a lack of growth for Black applicants, students, and graduates (14, 15). Understanding the factors that impact matriculation and persistence among current Black medical students may be beneficial to medical schools looking to increase the enrollment and graduation of Black students. Participants in this study reported their barriers to matriculation into medical school were a lack of exposure and a lack of resources. Exposure was related to a lack of exposure to someone in medicine while resources were related to things they needed to achieve their goals. The findings from this study on a lack of exposure being a barrier are similar to research on diversity in neurology which found that among Black medical students, a lack of exposure was the second reason for not choosing neurology as a specialty behind having a strong interest in another field (16). A lack of exposure to medicine was also cited by Black high school students as a barrier to pursuing a career in medicine (17). This study also reported financial challenges as a barrier to Black high school students not pursuing medicine which is similar to our findings related to a lack of resources, particularly financial resources, being a barrier to medical school (17).

Participants also reported that factors impacting their persistence once in medical school were diversity, support, and emotional resources. Diversity was related to the importance of diversity; support was related to having assistance or someone to lean on during difficult times; and emotional was related to having support from family, friends, partner, faculty/staff that they can lean on who keep them level-headed. The idea of diversity being important for persistence through medical school is similar to findings from a study looking at matriculation and graduation among minority medical students. While the findings were not statistically significant, the study reported that if minority students perceived they had enough racial/ethnic minority faculty members at their school, they had higher odds of graduating in 4 years than minority students who did not perceive they had enough racial/ethnic minority faculty (16). Additionally, the study reported statistically significant findings that minority students who had adequate mentorship had higher odds of graduating in 4 years compared to minority students who did not have adequate mentorship (16). Mentorship being important for graduating in 4 years for minority medical students is similar to our findings of having emotional support from faculty/staff being important for persistence while in medical school. Minority students who reported having a strong support system also had higher odds of graduating in 4 years compared to those without a strong support system (16).

This study revealed that barriers to matriculation are not the only factors that greatly affect the medical education of Black medical students. Findings show that a student’s path is influenced by experiences while in medical school, so access to a diverse and supportive environment can greatly improve a student’s success and emotional well-being. Institutions can use this information to establish programs and funding that can enhance a Black medical student’s experience by promoting an inclusive and welcoming environment. While providing a diverse faculty and staff is part of creating an inclusive and welcoming environment, medical schools should also work on how to make their curriculum, programming, and policies more inclusive and welcoming for Black students.

Future research in this area should approach this topic from the perspectives of undergraduate Black students interested in pursuing medicine, Black faculty within medical schools, family members of Black medical students, Black residents, and Black physicians. Having the perspectives of these different groups whom this study and other research have shown have an influence and impact on matriculation and persistence can greatly improve the scope of assessing the problem which can lead to a better understanding of this issue which can lead to more Black medical students in the future.

## Limitations

While this study included more than 16 medical schools in the US and Canada, most participants came from universities in the southern region and on the east coast; thus, findings may not be generalizable to all Black medical students. Additionally, most participants were first-year medical students who were in their first semester of medical school; thus, the findings may not have been as full or complete since these participants did not have a wealth of experiences yet in their medical school journey. Along this vein, most participants were not first-generation students so the findings may not be reflective of these students. However, being a Black medical student makes one a minority within a minority group that the authors do not believe the findings would have been drastically different if more participants were first-generation. Lastly, recruiting participants by reaching out to current medical students and medical school diversity offices may have accounted for participants coming from certain regions of the US. The sample may have been more representative of all Black medical students had a listserv been used; however, the first author had difficulties with that so convenience sampling was used. However, the similarity of responses across interviews shows that saturation of the data was reached and it is believed that findings would have been the same with a wider sample or more participants.

## Data availability statement

The datasets presented in this article are not readily available because the data is qualitative, it is unable to be provided. IRB approval does not cover making the data available to others. Requests to access the datasets should be directed to KM, mincey_kd@mercer.edu.

## Ethics statement

The studies involving human participants were reviewed and approved by Mercer University. Written informed consent for participation was not required for this study in accordance with the national legislation and the institutional requirements. Verbal informed consent was obtained from all participants.

## Author contributions

KM conceptualized the research, conducted the analysis, and wrote the paper. BR assisted with the analysis and writing of the paper. RJ assisted with the analysis and writing of the paper. MH assisted with the analysis. All authors contributed to the article and approved the submitted version.

## Funding

This research was supported by funding from Mercer University School of Medicine SEED grant.

## Conflict of interest

The authors declare that the research was conducted in the absence of any commercial or financial relationships that could be construed as a potential conflict of interest.

## Publisher’s note

All claims expressed in this article are solely those of the authors and do not necessarily represent those of their affiliated organizations, or those of the publisher, the editors and the reviewers. Any product that may be evaluated in this article, or claim that may be made by its manufacturer, is not guaranteed or endorsed by the publisher.
